# Eating High Fat Chow Decreases Dopamine Clearance in Adolescent and Adult Male Rats but Selectively Enhances the Locomotor Stimulating Effects of Cocaine in Adolescents

**DOI:** 10.1093/ijnp/pyv024

**Published:** 2015-04-08

**Authors:** Michelle G. Baladi, Rebecca E. Horton, William A. Owens, Lynette C. Daws, Charles P. France

**Affiliations:** Departments of Pharmacology (Drs Baladi, Daws, and France), Psychiatry (Dr France), and Physiology (Ms. Horton, Mr. Owens, and Dr. Daws), University of Texas Health Science Center at San Antonio, San Antonio, Texas

**Keywords:** Cocaine, locomotor activity, dopamine transporter, high fat chow, adolescents

## Abstract

**Background:**

Feeding conditions can influence dopamine neurotransmission and impact behavioral and neurochemical effects of drugs acting on dopamine systems. This study examined whether eating high fat chow alters the locomotor effects of cocaine and dopamine transporter activity in adolescent (postnatal day 25) and adult (postnatal day 75) male Sprague-Dawley rats.

**Methods:**

Dose-response curves for cocaine-induced locomotor activity were generated in rats with free access to either standard or high fat chow or restricted access to high fat chow (body weight matched to rats eating standard chow).

**Results:**

Compared with eating standard chow, eating high fat chow increased the sensitivity of adolescent, but not adult, rats to the acute effects of cocaine. When tested once per week, sensitization to the locomotor effects of cocaine was enhanced in adolescent rats eating high fat chow compared with adolescent rats eating standard chow. Sensitization to cocaine was not different among feeding conditions in adults. When adolescent rats that previously ate high fat chow ate standard chow, sensitivity to cocaine returned to normal. As measured by chronoamperometry, dopamine clearance rate in striatum was decreased in both adolescent and adult rats eating high fat chow compared with age-matched rats eating standard chow.

**Conclusions:**

These results suggest that high fat diet-induced reductions in dopamine clearance rate do not always correspond to increased sensitivity to the locomotor effects of cocaine, suggesting that mechanisms other than dopamine transporter might play a role. Moreover, in adolescent but not adult rats, eating high fat chow increases sensitivity to cocaine and enhances the sensitization that develops to cocaine.

## Introduction

Drug use during adolescence often predicts an increased likelihood of continued use into adulthood (Young et al., 2002; Merline et al., 2004; Arteaga et al., 2010). For example, adolescents who tried illicit drugs by their senior year of high school had a 5-fold greater likelihood of using cocaine at age 35 years compared with those who did not try illicit drugs by their senior year (Merline et al., 2004). Moreover, in 2012 there were 639,000 persons aged 12 years or older who had used cocaine for the first time within the past 12 months, corresponding to an average of approximately 1800 initiates per day (Substance Abuse and Mental Health Services Administration, 2013). Factors contributing to increased vulnerability to use drugs, including cocaine, during adolescence are not well understood but are thought to include social, economic, hormonal (e.g., insulin), neurochemical, and dietary conditions that influence individual responses to drugs (Spear, 2000; Baladi et al., 2012a; Daws et al., 2011; Niswender et al., 2011). Indeed, with the increasing high fat “fast-food” culture and prevalence of obesity, particularly in adolescents, diet might play a greater role than previously thought in determining the sensitivity of an individual to drugs, as well as predisposition to drug abuse (Baladi et al., 2012a; Wise, 2012; Volkow et al., 2013).

Cocaine increases extracellular dopamine (DA) by blocking DA uptake via the high-affinity DA transporter (DAT) (Kalivas and Duffy, 1990; Weiss et al., 1992; Giros et al., 1996; Amara et al., 1998). Converging lines of evidence suggest that insulin signaling is an important regulator of DAT, the primary target of cocaine (Figlewicz et al., 1994; Patterson et al., 1998; Owens et al., 2005; Sevak et al., 2007; Williams et al., 2007; Daws et al., 2011; Niswender et al., 2011; Speed, et al., 2011; Owens et al., 2012). Insulin receptors are coexpressed extensively with tyrosine hydroxylase (a marker for DA neurons; Hill et al., 1986; Figlewicz et al., 2003), and several studies have shown that insulin, via the phosphoinositide 3-kinase/Akt signaling pathway (Williams et al., 2007; Speed et al., 2011), regulates the expression and activity of DAT. Whereas insulin enhances DAT activity (Knusel et al., 1990; Carvelli et al., 2002), hypoinsulinemia (produced by experimentally induced diabetes, insulin resistance, or fasting) reduces DAT activity (Owens et al., 2005; Williams et al., 2007; Sevak et al., 2008; Speed et al., 2011; Owens et al., 2012). Moreover, dietary conditions that alter circulating insulin concentrations (Shiraev et al., 2009) can dramatically influence the behavioral effects of drugs acting on DA systems (Carroll et al., 1981; Sevak et al., 2008; Baladi and France, 2009, 2010). For example, rats eating high fat chow are significantly more sensitive than rats eating standard chow to the effects of drugs acting directly at D2 and D3 receptors (Baladi and France, 2009, 2010), and food-restricted rats are particularly sensitive to effects that are mediated by D2 receptors (Collins et al., 2008; Sevak et al., 2008). However, the consequences of eating high fat chow on the behavioral effects of cocaine are less clear, particularly with regard to whether there is an interaction between diet and age. A previous study (Baladi et al., 2012b) found that eating high fat chow increases the sensitivity of adolescent and adult female rats to cocaine and facilitates the development of sensitization to cocaine.

The aim of this study was to determine the effects of eating high fat chow on the locomotor-stimulating effects of cocaine and on DAT activity in striatum in adolescent and adult male rats. In experiment 1, rats ate either standard or high fat chow for either 1 or 4 weeks and then were tested once with cocaine. It was hypothesized that eating high fat chow would increase sensitivity of rats to the acute effects of cocaine based on the evidence that rats eating high fat chow have increased sensitivity to drugs acting directly at D2 and D3 receptors (Baladi and France, 2009, 2010), and these DA receptor subtypes indirectly mediate some of the behavioral effects of cocaine (McKenna and Ho, 1980; Spealman et al., 1991; Caine and Koob, 1993; Acri et al., 1995; Spealman, 1996; Kita et al., 1999). Furthermore, it is well known that sensitivity to some effects of cocaine increases over repeated testing (i.e., sensitization; Kalivas et al., 1993a, 1993b; Nestler, 1993; Izenwasser et al., 1999), and previous results indicate that eating high fat chow enhances the sensitization that develops to the locomotor effects of methamphetamine (McGuire et al., 2011). Thus, experiment 2 extends the generality of this finding by testing with cocaine once per week for 5 weeks in adolescent and adult rats eating either standard or high fat chow. Lastly, to determine whether diet-induced changes in sensitivity to the locomotor effects of cocaine are related to diet-induced changes in activity of DAT, DA clearance was measured from extracellular fluid in striatum. It has previously been shown that insulin resistance, caused by eating a high-fat diet, decreases DAT plasma membrane expression and activity in adult rats (Speed et al., 2011); thus, it was hypothesized that eating high fat food would similarly decrease DAT activity in adolescent rats.

## Materials and Methods

### Subjects

A total of 152 male Sprague-Dawley rats (Harlan, Indianapolis, IN) were used in the study. Seventy-eight adolescent rats at postnatal day (PND) 19 to 21 weighed 80 to 85g upon arrival, and 76 adult rats at PND 68 to 72 weighed 250 to 300g upon arrival; all rats were housed individually in an environmentally controlled room (24±1^o^C, 50 ±10% relative humidity) under a 12-h-light/-dark cycle with water available continuously. Rats had either free or restricted access to chow (see below), except during experimental sessions when no food was available (for all rats). Animals were maintained and experiments were conducted in accordance with the Institutional Animal Care and Use Committee, the University of Texas Health Science Center at San Antonio, and with the *Guide for Care and Use of Laboratory Animals* (Institute of Laboratory Animal Resources).

### Feeding Conditions

**Figure 1.**
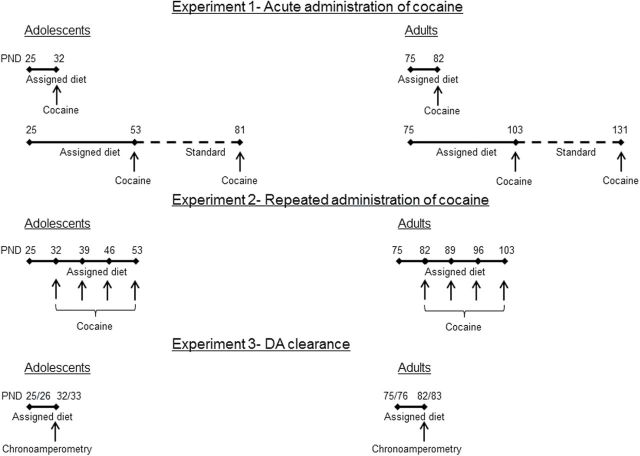
Experimental design. Numbers above time lines indicate age as postnatal day (PND) and slash mark between numbers represent the range of PND for groups (e.g., PND 25/6). Top time line is experiment 1 (acute administration of cocaine and locomotor activity), middle time line is experiment 2 (repeated administration of cocaine and locomotor activity), and bottom time line is experiment 3 (dopamine [DA] clearance).

Rats were fed either standard or high fat chow beginning at either PND 25/26 (for adolescents) or PND 75/76 (for adults) and according to the conditions described below (also see Figure 1). The nutritional content of the standard chow (Harlan Teklad 7912) was 5.7% fat, 44.3% carbohydrate, and 19.9% protein (by weight), with a calculated gross energy content of 4.1 kcal/g. The high fat chow (Harlan Teklad 06414) contained 34.3% fat, 27.3% carbohydrate, and 23.5% protein (by weight), with a calculated energy content of 5.1 kcal/g. Body weights were measured daily for all rats.

In experiment 1, a single test with cocaine was conducted after adolescent and adult rats (n = 6/feeding condition) had free access to either standard or high fat chow or restricted access (adjusted daily to match body weights of rats with free access to standard chow) to high fat chow for either 1 or 4 weeks. Next, all rats in experiment 1 had free access to standard chow for 4 weeks followed by a second test with cocaine. In experiment 2 (Figure 1), tests with cocaine occurred once per week for 5 weeks. Initially, all rats in experiment 2 had free access to standard laboratory chow; after a first test with cocaine, adolescent and adult rats either continued to have free access to standard chow, free access to high fat chow, or restricted access to high fat chow for 4 weeks (n = 6/feeding condition). In experiment 3 (Figure 1), DA clearance was studied using chronoamperometry in adolescent (n= 7–8/feeding condition) and adult (n = 7–8/feeding condition) rats that had free access to either standard or high fat chow or restricted access to high fat chow for 1 week.

### Apparatus

Four Plexiglass chambers, measuring 26×61×23cm high (Instrumentation Services, The University of Texas Health Science Center, San Antonio, TX) and located within ventilated sound-attenuating cubicles (MED Associates Inc., St. Albans, VT), were used to assess locomotor activity (Owens et al., 2005). Two of the chambers had a metal floor with holes (6-mm diameter, 9mm center to center), and the other 2 chambers had a metal grid floor (6×6-mm wire mesh supported by 5-mm-diameter metal rods spaced 16mm center to center). For experimental sessions, one-half of the rats in each group were studied in the chambers that had a metal floor with holes and the other one-half in the chambers that had a metal grid floor. Preliminary data showed that basal locomotor was not significantly different between floor textures, and individual rats were always studied in the same chamber with the same floor texture. Horizontal activity in the chamber was measured with 4 pairs of infrared photo beams (Multi-Varimex, Columbus Instruments, Columbus, OH) positioned 4cm above the floor of the chamber. The photo beams were separated by 15cm with 2 photo beams located 8cm from the ends of the chamber.

### Locomotor Activity

At the beginning of experiment 1, adolescent (n=36) and adult (n=36) rats with free access to standard chow were acclimated to the locomotor chamber for 120-minute periods on each of 2 days (beginning 30 minutes after placement in the chamber, saline was injected every 15 minutes for a total of 6 injections). After the 2-day acclimation period, all adolescent and adult rats were tested again with saline then assigned to their respective feeding condition at PND 25 or 75, respectively. After either 1 (PND 32 or 82) or 4 (PND 53 or 103) weeks on these feeding conditions, adolescent and adult rats (n= 6/feeding condition) were tested once with cumulative doses of cocaine (5.6–56.0mg/kg; i.p.). Two days after the test with cocaine, all rats were again tested with saline to determine whether acute testing with cocaine or eating high fat chow changed basal locomotion (compared with locomotion before cocaine tests and when all rats had free access to standard chow; basal locomotor data were not different across the study). Next, the 36 rats (18 adolescents and 18 adults) in experiment 1 that were tested with cocaine after 4 weeks on a particular diet were given free access to standard chow for an additional 4 weeks prior to redetermination of a second cocaine dose-response curve (PND 81 and 131, respectively). Initially, adolescent (n=18) and adult (n=18) rats in experiment 2 had free access to standard chow and were acclimated to the locomotor chamber as described above for experiment 1. After the acclimation period, rats were tested with cumulative doses of cocaine (1.0–17.8mg/kg; i.p.) at PND 25 or 75. Tests with cocaine were identical to experiment 1 except that smaller doses were studied and rats received only 5 injections in a session. After an initial cocaine test, adolescent and adult rats were assigned to their respective feeding condition (on PND 25 or 75, respectively; n= 6/feeding condition) and thereafter were tested with cocaine once per week for 4 weeks. Two days after the last cocaine test, all rats were tested again with saline to determine whether repeated testing with cocaine or eating high fat chow changed basal locomotion (basal locomotor data were not different across the study).

### In Vivo Chronoamperometric Recordings of DA Clearance

Chronoamperometry was performed in adolescent (n=7–8/feeding condition) and adult (n=6–8/feeding condition) rats that had free access to either standard or high fat chow or restricted access to high fat chow for 1 week immediately prior to testing (Figure 1). Adolescent rats were assigned to their respective feeding condition on PND 25 or 26 (i.e., testing occurred on PND 32 or 33). Similarly, adult rats were assigned to their respective feeding condition on PND 75 or 76 (i.e., testing on PND 82 or 83). Clearance of exogenously applied DA was measured by high-speed chronoamperometry using the FAST-12 system (Quanteon, Nicholasville, KY) as described elsewhere (Owens et al., 2005, 2012; Speed et al., 2011). Oxidation potentials consisted of 100-ms pulses of +0.55V with each pulse separated by 900ms during which resting potential was held at 0.0V. Carbon fiber electrodes were coated with Nafion (5%) to prevent interference from anionic substances in the extracellular fluid. Electrodes were tested for sensitivity to the DA metabolite 3,4-dihydroxyphenylacetic acid (DOPAC, 20 μM). Using DOPAC as the challenge compound, all electrodes were calibrated in vitro with increasing concentrations of DA in 1.5-μM increments. Only electrodes displaying a selectivity ratio >1000:1 (i.e., DA:DOPAC) were used. Rats were anesthetized by chloralose (85mg/kg, i.p.) and urethane (850mg/kg, i.p.) and prepared for chronoamperometry (Owens et al., 2005). The electrode-micropipette recording assembly was lowered into the dorsal striatum (AP, +1.2; ML, + 2.2; DV, -3.5 to -5.5; Paxinos and Watson, 1986). The dorsal striatum is a region rich in DAT expression and DA signaling in this region is critical for feeding behavior (Sotak et al., 2005). In addition, the dorsal striatum plays a role in mediating the locomotor effects of DA receptor agonists (McDougall et al., 1992). Barrels were filled with DA (200 μM) dissolved in 0.1M phosphate buffered saline with 100 μM ascorbic acid added as an antioxidant (pH 7.3–7.4). DA was pressure ejected at 5-minute intervals in randomized order among rats (see also Zahniser et al., 1999). Other than an anesthetic, no drug was present at the time of chronoamperometric measurements.

### Data Analyses

For locomotor activity experiments, data for 5-minute periods beginning 10 minutes after injections are expressed as mean (±SEM) activity counts for each group and plotted as a function of dose. A 2-way (dose and feeding condition), repeated-measures ANOVA with posthoc Bonferroni’s test was used to determine whether cocaine-induced locomotor activity in adolescent or adult rats with free or restricted access to high fat chow was significantly different from activity in adolescent or adult rats with free access to standard chow (GraphPad Prism; GraphPad Software Inc., San Diego, CA). Chronoamperometry data were analyzed using the following 3 signal parameters: (1) the maximal amplitude (in μM) of the signal; (2) clearance rate (in nM/s), defined as the slope of the decay curve from 20% to 60% of maximal signal amplitude (i.e., the approximately linear portion of the decay); and (3) T_80_, the time (s) for the signal to decline by 80% of the maximal amplitude. The effects of feeding condition on T_80_ and signal amplitude for 80 pmol DA in adolescent rats and 100 pmol DA in adult rats were analyzed using a 1-way ANOVA (feeding condition). The mean area under the curve (AUC; ±SEM) was also calculated for each cocaine dose-response curve and for chronoamperometry functions. A 1-way, repeated-measures ANOVA was used to determine whether the AUC in adolescent or adult rats with free or restricted access to high fat chow was significantly different from the AUC in adolescent or adult rats with free access to standard chow (GraphPad Prism), respectively; posthoc multiple comparisons were made with the Dunnett’s test. For all tests, significance was set at *P*<.05.

### Drugs

Cocaine was provided by the Research Technology Branch, National Institute on Drug Abuse (Rockville, MD) and dissolved in sterile 0.9% saline. DA hydrochloride, DOPAC, ascorbic acid, Nafion (5%), chloralose, and urethane were purchased from Sigma-Aldrich (St. Louis, MO). Chloralose, urethane, DA, DOPAC, and ascorbic acid were dissolved in 0.1M phosphate buffered saline. Drug and saline were administered i.p. in a volume of 1mL/kg.

## Results

### Experiment 1: Acute Administration of Cocaine

**Figure 2.**
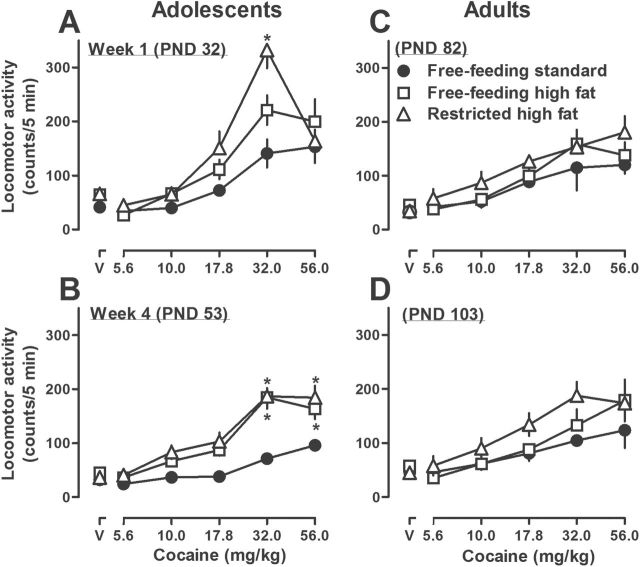
Effects of acutely administered cocaine on locomotor activity in adolescent (A-B) and adult (C-D) rats with free access to either standard or high fat chow or restricted access to high fat chow for either 1 (A,C) or 4 (B,D) weeks. Each condition represents the mean ±SEM of 6 rats (i.e., data from different groups of rats are presented in different panels with symbols designating feed condition). Abscissae: dose in mg/kg of body weight; data points above V indicate the effects obtained with vehicle. Ordinates: mean locomotor activity counts/5min (±SEM). *P<.05 compared with rats that ate standard chow throughout the study at the corresponding dose of cocaine and analyzed by a 2-way (dose and feeding condition), repeated-measures ANOVA with posthoc Bonferroni’s test.

**Figure 3.**
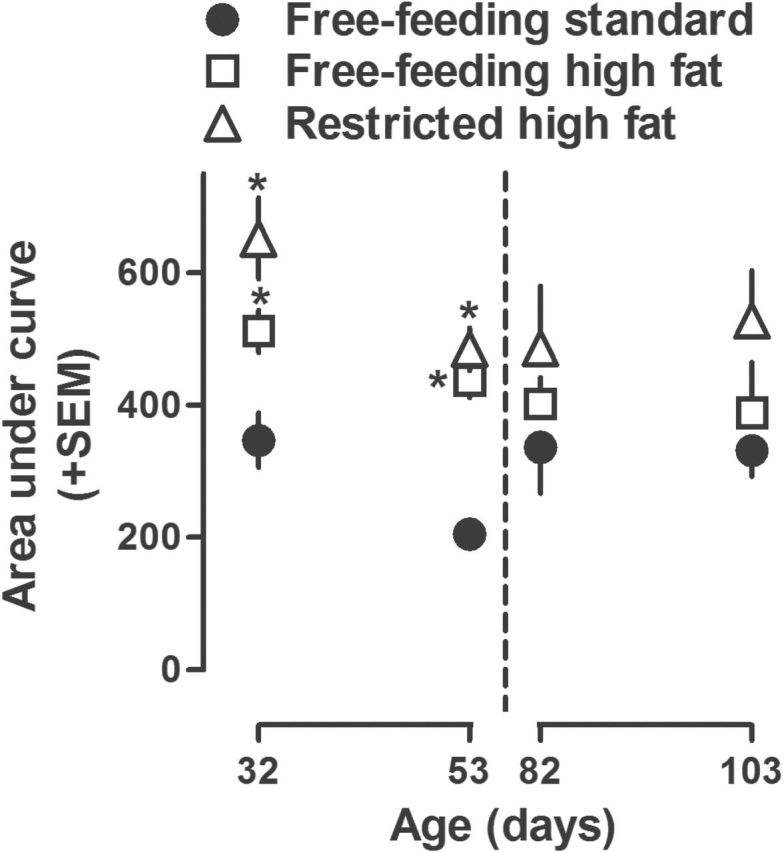
Area under the curve (AUC) in adolescent (postnatal day [PND] 32 and 53, left panel) and adult (PND 82 and 103, right panel) rats with free access to either standard or high fat chow or restricted access to high fat chow for either 1 or 4 weeks and treated acutely with cocaine. Each symbol represents the mean ±SEM of 6 rats (same data as plotted in Figure 1). Abscissa: PND age in days. Ordinate: mean AUC (±SEM).*P<.05 compared with rats that had free access to standard chow at the corresponding age and analyzed by a 1-way (feeding condition), repeated-measures ANOVA with posthoc Bonferroni’s test.

Cocaine increased locomotor activity in a dose-related manner in adolescent and adult rats, with maximum increases occurring at cumulative doses of 32 and 56mg/kg, respectively (left and right panels, Figure 2). Adolescent rats with restricted access to high fat chow for 1 week showed a greater locomotor response to cocaine compared with rats eating standard chow (Figure 2A). For example, the average locomotor activity at a dose of 32.0mg/kg cocaine was 332±33 in rats with restricted access to high fat chow and 141±26 in rats with free access to standard chow. Compared with adolescent rats eating standard chow for 4 weeks, locomotor effects of cocaine were significantly greater, at doses of 32.0 and 56.0mg/kg cocaine, in adolescent rats with free or restricted access to high fat chow (Figure 2B). Data from cocaine dose-response curves in Figure 2 are replotted as AUC (±SEM) in Figure 3 and show a significant increase in adolescent rats eating high fat chow for 1 (PND 32) or 4 (PND 53) weeks. The body weights were not significantly different among the 3 groups of adolescent rats (Table 1). For example, after 4 weeks, the average body weights (±SEM) were 255±11g for rats with free access to standard chow, 250±14g for rats with restricted access to high fat chow (feeding was adjusted so as to match their body weights to rats eating standard chow), and 268±13g for rats with free access to high fat chow.

**Table 1.** Body Weights of Adolescent and Adult Rats

Week	Free-Feeding Standard	Restricted High Fat	Free-Feeding High Fat
Adolescents			
Experiment 1			
1	106 (±3)^a^	107 (±4)	106 (±4)
4	255 (±11)	250 (±14)	268 (±13)
8	362 (±9)	367 (±4)	365 (±2)
Experiment 2			
1	134 (±2)	134 (±3)	141 (±4)
4	271 (±8)	272 (±5)	288 (±8)
Experiment 3			
1	106 (±2)	108 (±3)	104 (±2)
Adults			
Experiment 1			
1	322 (±6)	323 (±6)	326 (±6)
4	371 (±6)	376 (±4)	393 (±4)^b^
8	417 (±4)	427 (±7)	449 (±7)^b^
Experiment 2			
1	329 (±2)	329 (±4)	346 (±4)
4	371 (±6)	376 (±4)	398 (±6)^b^
Experiment 3			
1	334 (±11)	333 (±5)	345 (±4)

^*a*^Values represent the mean (±SEM) body weight in grams.

^*b*^Values are significantly different compared to the respective age-matched group eating standard chow, *P*<.05.

In contrast, the locomotor-stimulating effects of cocaine were not different among adult rats eating standard or high fat chow for either 1 or 4 weeks (Figure 2C-D). Moreover, AUC for dose-response curves in Figure 2 was not different among feeding conditions at either 1 (PND 82) or 4 (PND 103) weeks (Figure 3). While body weight was not different between rats with free access to standard chow and those with restricted access to high fat chow (371±6 and 376±4g, respectively, in week 4), body weight was significantly increased in adult rats with free access to high fat chow (e.g., 393±4g in week 4; Table 1). Of note, there was no significant difference between adolescent and adult rats eating standard chow with regard to the locomotor effects of acutely administered cocaine (Figure 3, compare circles). For rats eating standard chow, the AUC in Figure 3 across age was 347±41, 205±14, 336±69, and 332±39 at PND 32, 53, 82, and 103, respectively.

**Figure 4.**
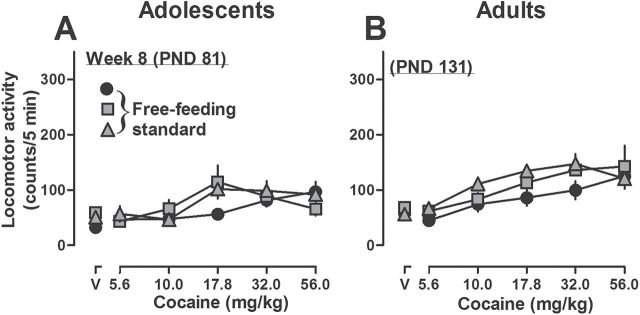
Effects of acutely administered cocaine on locomotor activity in adolescent (A) and adult (B) rats that ate standard chow for 4 weeks. Circles indicate rats that ate standard chow throughout the study; gray symbols indicate rats that previously had free (squares) or restricted (triangles) access to high fat chow (n=6 per group). See Figure 2 for other details.

When adolescent rats that ate high fat chow for 4 weeks were given free access to standard chow for 4 weeks, the locomotor effects of cocaine decreased and were not different from the effects of cocaine in rats that ate only standard chow (i.e., PND 81) (Figure 4A). After eating standard chow for 4 weeks, body weight was not significantly different among the 3 groups of adolescent rats (Table 1). AUCs for dose-response curves shown in the left panel of Figure 4 were not different among groups (data not shown). Similarly, when adult rats that ate high fat chow for 4 weeks were given free access to standard chow for 4 weeks, the locomotor effects (cocaine dose-response curves; Figure 4B) as well as the AUCs (data not shown) were not different among the 3 groups. After eating standard chow for 4 weeks, the body weight of rats that previously ate high fat chow remained significantly higher (449±7g) compared with rats that ate only standard chow (417±4g; Table 1).

### Experiment 2: Repeated Administration of Cocaine

**Figure 5.**
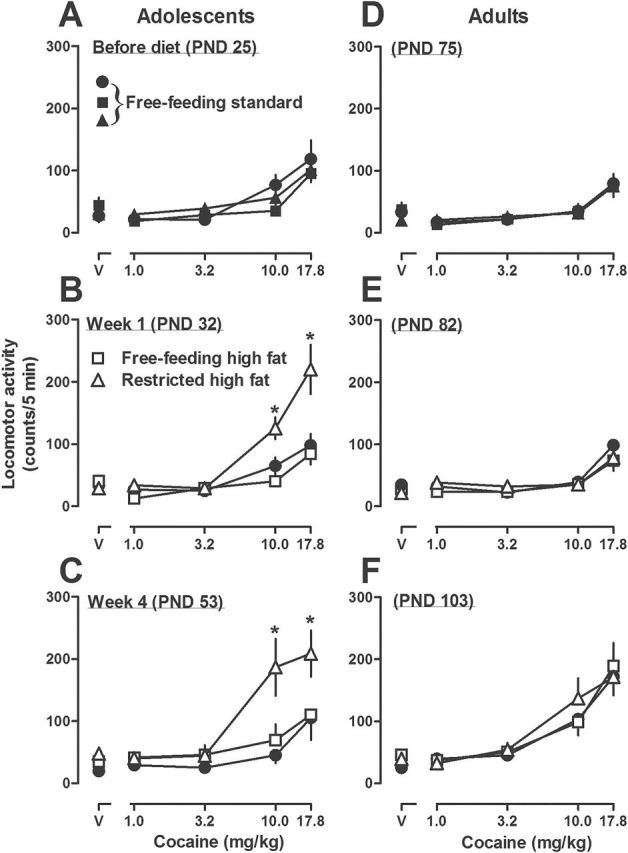
Effects of repeated cocaine administration on locomotor activity in adolescent (A-C) and adult (D-F) rats with free access to standard chow (A,D) followed by free access to either standard or high fat chow or restricted access to high fat chow for 1 (B,E) or 4 (C,F) weeks (data from the same rats [adolescent left, adult right] are presented in upper, middle, and lower panels; n=6 per group). See Figure 2 for other details.

Cocaine increased locomotor activity in adolescent (PND 25) rats eating standard chow (Figure 5A). The effects of cocaine were significantly increased in rats with restricted access to high fat chow for 1 week (10.0 and 17.8mg/kg; Figure 5B); this difference between rats with restricted access to high fat chow and those with free access to either high fat or standard chow was still evident after 3 additional once-weekly tests with cocaine (i.e., week 4, PND 53, Figure 5C). The effects of cocaine were not significantly different between rats with free access to standard chow or free access to high fat chow (Figure 5B-C; data not shown for weeks 2 and 3). Body weight was not significantly different at any week among the 3 groups of adolescent rats (Table 1).

**Figure 6.**
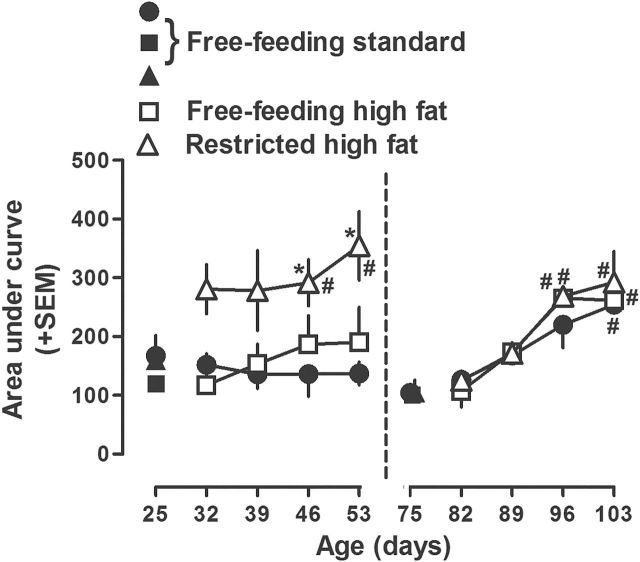
Area under the curve (AUC) in adolescent (postnatal day [PND] 25–53, left panel) and adult (PND 75–103, right panel) rats with free access to either standard or high fat chow or restricted access to high fat chow and tested once per week with cocaine for 4 weeks. Each symbol represents the mean ±SEM of 6 rats (same data as plotted in Figure 4). Abscissa: PND age in days. Ordinate: mean AUC (±SEM). *P<.05 compared with rats that had free access to standard chow throughout the study at the corresponding age. #*P*<.05 compared with the same rats when they had free access to standard chow at the beginning of the study (i.e., PND 25 or 75).

Data from cocaine dose-response curves in the left panels (A-C) of Figure 5 are replotted as AUC in the left panel of Figure 6 and show a significant increase in weeks 3 and 4 (PND 46 and 53) in rats with restricted access to high fat chow compared with the effects of cocaine in the same rats prior to access to high fat chow and compared with the effects of cocaine in weeks 3 and 4 in rats eating standard chow. There was no significant difference in AUC between rats eating standard chow and those with free access to high fat chow and no significant difference across once weekly testing with cocaine for either of those groups of rats.

Cocaine increased locomotor activity in adult (PND 75) rats eating standard chow (Figure 5D). The effects of cocaine were not significantly different among adult rats that ate standard or high fat (free or restricted access) chow for 1 or 4 weeks (Figure 5E and F, respectively; data not shown for weeks 2 and 3). While body weight was not significantly different between rats with free access to standard chow and those with restricted access to high fat chow, body weight was significantly increased in rats with free access to high fat chow compared with rats eating standard chow (371±6g and 398±6g, respectively, in week 4; Table 1). Data from cocaine dose-response curves in the right panels (D-F) of Figure 5 are replotted as AUC in the right panel of Figure 6 and show a significant increase across weeks for all 3 groups of rats, although there was no difference among groups for any test. Finally, it is noteworthy that sensitization to cocaine developed in adult, but not adolescent, rats eating standard chow (Figure 6, compare circles).

### Experiment 3: DA Clearance

**Figure 7.**
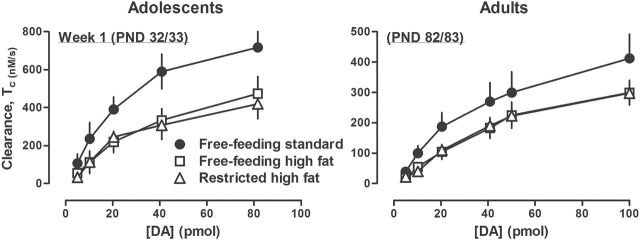
Rate of dopamine (DA) clearance (nM/sec) in adolescent (A) and adult (B) rats with free access to either standard or high fat chow or restricted access to high fat chow for 1 week. Each symbol represents the mean ±SEM of 6 to 8 rats. Abscissa: concentration of DA in pmol. Ordinates: mean DA clearance rate in nM/sec (±SEM).

The rate of DA clearance in the striatum was decreased in adolescent (PND 32 or 33) rats eating high fat chow (free or restricted access) compared with adolescent rats eating standard chow (Figure 7A). The AUC (±SEM) for the rate of DA clearance was 902±116 for rats with free access to high fat chow, 877±104 for rats with restricted access to high fat chow, and 1582±271 for rats with free access to standard chow. The effect of eating high fat chow (free or restricted access) was similar in adult rats, with the rate of DA clearance in striatum of adult (PND 82 or 83) rats eating high fat chow being decreased compared with rats eating standard chow (Figure 7B). The AUC for the rate of DA clearance was 666±116 for adult rats with free access to high fat chow, 552±69 for adult rats with restricted access to high fat chow, and 1280±124 for adult rats with free access to standard chow. Differences in the rate of DA clearance in both adolescent and adult rats were accompanied by longer T_80_ values in rats eating high fat chow compared with rats eating standard chow (Table 2). Furthermore, and as expected, DA signal amplitude increased with increasing pmol amounts of DA pressure-ejected into dorsal striatum (range approximately 0.5 to 25.0 μM). However, DA signal amplitude per pmol of DA pressure-ejected did not differ between age groups or among feeding conditions (data not shown). Body weight was not significantly different among the 3 groups of adolescent rats (Table 1). Lastly, there was no significant difference between adolescent and adult rats eating standard chow with regard to the effects of age on DA clearance rate (Figure 7, compare circles between panels). That is, the AUC for DA clearance rate was 1582±271 for adolescent rats with free access to standard chow and 1280±124 for adult rats with free access to standard chow.

**Table 2.** Clearance Time (T80, in s) for DA (40 pmol) in Striatum of Adolescent and Adult Rats across Feeding Conditions

Feeding Condition	N	Adolescent	Adult
Free-feeding standard	8	27.4 (±3.5)^*a*^	29.3 (±3.3)
Free-feeding high fat	8	39.0 (±4.3)^*b*^	41.8 (±4.9)^*b*^
Restricted high fat	7	36.2 (±2.7)^*b*^	44.4 (±4.8)^*b*^

^*a*^Values represent the mean (±SEM) for the number of animals indicated. All rats ate their respective chow for 1 week.

^*b*^Values are significantly different compared to the respective age-matched group eating standard chow, *P*<.05.

## Discussion

The first major finding of the current study is that eating high fat chow, in adolescent but not adult male rats, increases sensitivity to the locomotor stimulating effects of acutely administered cocaine and enhances the development of sensitization to repeatedly administered cocaine. These findings in male rats are in contrast to the effects reported for the same dietary conditions in female rats, whereby eating high fat chow in both adolescent and adult female rats increased sensitivity to acutely administered cocaine and facilitated the development of sensitization to repeatedly administered cocaine (Baladi et al., 2012b). Thus, and unlike female rats, it appears that eating high fat chow increases the behavioral effects of cocaine in male rats within a relatively specific age window occurring during adolescence, but not during adulthood. This increased sensitivity to the behavioral effects of acute or repeated cocaine in adolescent male rats might be related to increased vulnerability to drug abuse during adolescence. The second major finding of the current study is that the rate of DA clearance in striatum was decreased in both adolescent and adult rats eating high fat chow (regardless of free or restricted access) compared with adult rats eating standard chow. These results suggest that age- and diet-dependent differences in locomotor response to cocaine are not exclusively related to high fat diet-induced decreases in DAT activity, the primary target of cocaine in the striatum.

The underlying mechanism(s) whereby eating high fat chow might alter sensitivity to the locomotor effects of cocaine and DAT activity remains unclear. However, eating high fat chow can modify circulating concentrations of insulin, and insulin directly alters the expression and activity of DAT in a manner such that low concentrations of insulin decrease (Owens et al., 2005, 2012; Williams et al., 2007; Sevak et al., 2008; Speed et al., 2011) and high concentrations increase (Knusel et al., 1990; Carvelli et al., 2002) DAT activity. Although insulin levels were not measured in the current study, sustained consumption of high fat chow can lead to insulin resistance (Davidson and Garvey, 1993; Wilkes et al., 1998; Durham et al., 2006; Posey et al., 2009; Morris et al., 2011), and this effect of eating high fat chow on insulin sensitivity has been demonstrated in other studies using similar dietary conditions (Baladi et al., 2011; Speed et al., 2011). Importantly, high fat diet-induced insulin resistance also leads to decreased insulin signaling and a predicted decrease in DAT activity (Speed et al., 2011; Cone et al., 2013; Narayanaswami et al., 2013). Although a mechanistic link needs to be further validated between changes in insulin levels and DAT function, in the current study, DA clearance (i.e., DAT activity) was reduced in all rats eating high fat chow, regardless of age. Furthermore, these data are consistent with those reported by Speed et al. (2011), who showed that (1) insulin resistance, produced by eating high fat chow, decreases DAT plasma membrane expression and activity and (2) restoration of insulin signaling pathways (i.e., Akt) rescues high fat diet-induced decreases in DAT expression.

Compared with DAT and DA clearance, much less is known about the effects of reduced insulin signaling and eating high fat chow on DA receptor expression and function as well as extracellular DA concentrations (see Murzi et al., 1996; Ohtani et al., 1997; Rada et al., 2010). In addition, several other non-DA neurotransmitter systems (serotonin, opioid, and cannabinoid) as well as the hypothalamus are thought to regulate food intake (Volkow et al., 2011), and the role that these systems play in diet-induced changes in sensitivity to drugs is unclear. Nevertheless, and in regards to DA systems, depending on the duration that subjects were maintained on a particular dietary condition, studies have reported increases (South and Huang, 2008) or decreases (Narayanaswami et al., 2013) in DA receptor (i.e., D2) expression. Nevertheless, while decreased DA clearance (e.g., DAT activity) might initially increase extracellular DA concentration, long-term suppression of insulin signaling might lead to a reduction in extracellular DA (see Rada et al., 2010), compensatory upregulation of DA receptors (i.e., D1, D2, and D3; South and Huang, 2008) known to mediate many effects of cocaine, and thus increased sensitivity to the behavioral effects of drugs acting on those receptors.

That eating high fat chow did not increase sensitivity to cocaine in adult rats (as it did in adolescent rats) might be due to age-related differences in DA systems and/or the manner in which high fat chow impacts these systems. In rats, DA receptor density, particularly D1 and D2 receptors, increases between PND 25 and 40 (adolescent) then decreases between PND 40 and 120 (late adolescent to adult) (Andersen and Teicher, 2000). Whether DAT expression and/or activity varies across these ages is less clear, with studies reporting increased (Moll et al., 2000), decreased (Matthews et al., 2013), or no difference (Cao et al., 2007) in DAT expression between adolescent and adult rats. However, protein expression does not always strictly correlate with functional activity and, in this regard, the results are again mixed. For example, in vitro studies using striatal synaptosome preparations by Volz et al. (2009) demonstrated that adolescent rats have increased DAT activity compared with adult rats, whereas Kokoshka et al. (2000) found no difference in DAT activity between adolescent and adult rats. Results from the current in vivo study are in reasonable agreement with both reports. Although statistically significant differences were not reached, there was a clear trend for the rate of DA clearance to be faster in adolescent than in adult rats. Regardless of these reported differences in basal DAT activity between adolescent and adult rats, in the present study the effect of eating a high fat chow to reduce DA clearance did not directly predict locomotor response to cocaine. These results suggest that increased sensitivity of adolescent, but not adult, rats eating high fat chow to the locomotor effects of cocaine is likely related to the following: (1) age-dependent increased functional activity of postsynaptic DA receptors; and/or (2) diet-induced alterations to non-DA systems (e.g., serotonin receptors and transporter; Huang et al., 2004; du Bois et al., 2006) that might induce adaptive changes in mechanisms controlling DA neurotransmission (Carboni and Silvagni, 2004; Larsen et al., 2011) in a manner that differs between adolescent and adult rats.

One important aspect of the present study is that in adolescent rats, diet-induced changes in sensitivity to the acute effects of cocaine were not accompanied by differences in body weight. Thus, it is the type of chow (i.e., high fat) that adolescent rats eat, and not changes in body weight, which most likely contributes to their increased sensitivity to cocaine. Moreover, and as shown in other studies, not all effects of a drug change in a similar manner across feeding conditions, suggesting that changes in the locomotor effects of cocaine were not due to pharmacokinetic factors (Baladi et al., 2009, 2011). In general, food restriction can enhance the behavioral effects of cocaine in rats (Bell et al., 1997; Carr et al., 2001). Thus, restricting access to high fat chow might further contribute to increased sensitivity to cocaine, a notion supported by the present data but specifically for adolescents. For example, although adolescent rats with either restricted or free access to high fat chow were more sensitive than rats with free access to standard chow to the locomotor effects of cocaine, rats with restricted access to high fat chow were more sensitive than those with free access to high fat chow.

Repeated administration of cocaine can induce a progressive and enduring enhancement of locomotor stimulating effects (i.e., sensitization). Although there are a number of reports showing cocaine-induced sensitization in adult rats (Kalivas and Stewart, 1991; Henry and White, 1995; Hope et al., 2006), sensitization to cocaine does not appear to develop as robustly in adolescent rats (Collins and Izenwasser, 2002; Frantz et al., 2007; although see Marin et al., 2008). In addition, the development of drug-induced sensitization can depend on experimental conditions such as frequency and dose of drug administration. In the current study, cocaine-induced sensitization did not occur in adolescent rats with free access to either standard or high fat chow while restricted access to high fat chow enhanced the development of sensitization to cocaine. In adult rats, sensitization developed to cocaine but was not significantly different among feeding conditions. Although, in adult rats, eating high fat chow enhanced sensitization to the locomotor stimulating effects of methamphetamine (McGuire et al., 2011), the current findings suggest that whether eating high fat chow impacts drug-induced sensitization is dependent on age (i.e., adolescents vs adults), drug (i.e., methamphetamine vs cocaine), and access conditions (i.e., free vs restricted).

When adolescent rats that ate high fat chow were returned to eating standard chow, sensitivity to the locomotor stimulating effects of cocaine returned to control values (i.e., not different from rats that ate standard chow throughout the study). It is possible that longer access to high fat chow (i.e., more than 4 weeks) would have more enduring effects on sensitivity to cocaine even when rats are returned to standard chow. Several studies have shown that the younger rats are when they are exposed to some drugs, the greater their sensitivity is to the same drugs in adulthood (Kostrzewa et al., 1993; White and Holtzman, 2005). This finding might extend to food consumption such that earlier access to high fat chow might increase subsequent sensitivity to cocaine or related drugs (Naef et al., 2008; Shalev et al., 2010).

In summary, the current study demonstrates that eating high fat chow during adolescence significantly enhances sensitivity to acutely administered cocaine as well as to repeatedly administered cocaine (i.e., sensitization). This finding is sex-dependent, with both adolescent males (present study) and females (Baladi et al., 2012b) showing enhanced sensitivity to cocaine, whereas adult females (Baladi et al., 2012b), but not males (present study), also shared this enhanced response. Enhanced responses to the locomotor effects of cocaine were greatest when access to high fat chow was restricted. In contrast, both restricted and free access to high fat chow reduced DAT activity, indexed by decreased rates of DA clearance in striatum. This result suggests that enhanced sensitivity to the locomotor effects of cocaine is likely driven by age- and/or high fat diet-induced increases in functional activity of postsynaptic DA receptors, putatively D2-like receptors. Given the increasing high fat “fast-food” culture, particularly in adolescents, diet might play a greater role than previously thought in determining the sensitivity of an individual to drugs as well as predisposition to drug abuse.

## Statement of Interest

None.
